# Molecular Basis of Differential Stability and Temperature Sensitivity of ZIKA *versus* Dengue Virus Protein Shells

**DOI:** 10.1038/s41598-020-65288-3

**Published:** 2020-05-21

**Authors:** Chinmai Pindi, Venkat R. Chirasani, Mohammad Homaidur Rahman, Mohd Ahsan, Prasanna D. Revanasiddappa, Sanjib Senapati

**Affiliations:** Department of Biotechnology and BJM School of Biosciences, Indian Institute of Technology Madras, Chennai, 600036 India

**Keywords:** Computational biology and bioinformatics, Molecular modelling

## Abstract

Rapid spread of ZIKA virus (ZIKV) and its association with severe birth defects have raised worldwide concern. Recent studies have shown that ZIKV retains its infectivity and remains structurally stable at temperatures up to 40 °C, unlike dengue and other flaviviruses. In spite of recent cryo-EM structures that showed similar architecture of ZIKA and dengue virus (DENV) E protein shells, little is known that makes ZIKV so temperature insensitive. Here, we attempt to unravel the molecular basis of greater thermal stability of ZIKV over DENV2 by executing atomistic molecular dynamics (MD) simulations on the viral E protein shells at 37 °C. Our results suggest that ZIKA E protein shell retains its structural integrity through stronger inter-raft communications facilitated by a series of electrostatic and H-bonding interactions among multiple inter-raft residues. In comparison, the DENV2 E protein shell surface was loosly packed that exhibited holes at all 3-fold vertices, in close agreement with another EM structure solved at 37 °C. The residue-level information obtained from our study could pave way for designing small molecule inhibitors and specific antibodies to inhibit ZIKV E protein assembly and membrane fusion.

## Introduction

The recent spread of ZIKA virus and its association with microcephaly and Guillain-Barre syndrome have raised major concerns worldwide. Since its report in 2015 from Brazil, the virus has affected more than a million individuals across the world^1^. Consequently, in March 2016, WHO declared an international health emergency over ZIKA virus outbreak that caused serious birth defects^2^. The ZIKA virus is transmitted to humans primarily by mosquitoes, but reports of transmission through other means have also been documented recently^3,4^. The virus belongs to the family flaviviridae that also includes dengue, west nile, japanese encephalitis, and yellow fever viruses^5^.

**Figure 1:**
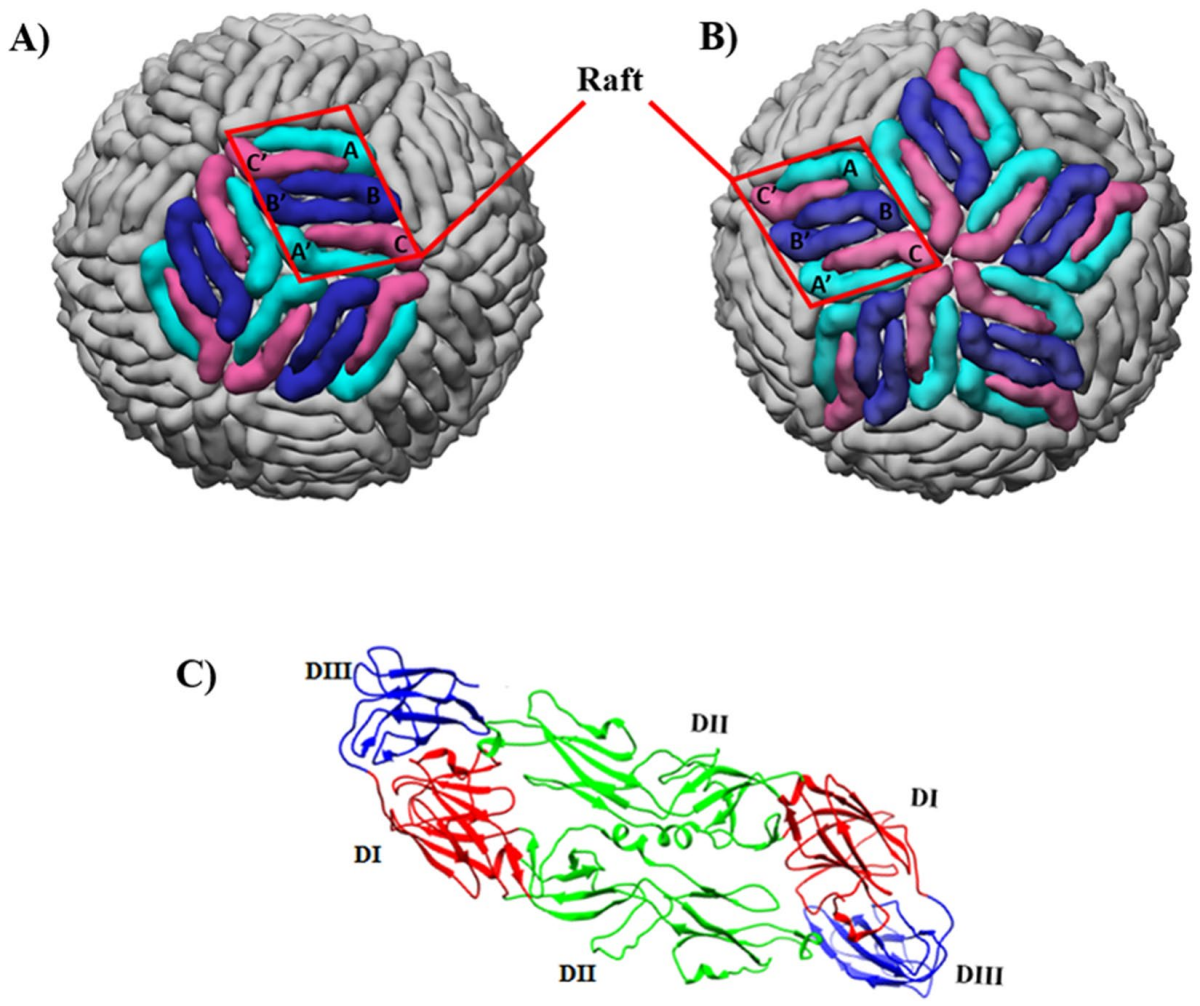
Architecture of the viral glycoprotein shell. On the shell surface, (**A**) a 3-fold vertex comprised of three intersecting rafts and (**B**) a 5-fold vertex comprised of five intersecting rafts, are highlighted. Conventional nomenclature of the six constituent E proteins in a raft is used with molecule A/A’ shown in cyan, molecule B/B’ in blue, and molecule C/C’ in pink. (**C**) E protein homodimer showing the constituent domains. The figures are generated by Chimera 1.11 software suite (http://www.rbvi.ucsf.edu/chimera)^30^ with the coordinates of ZIKV glycoprotein extracted from PDB (PDB ID: 5IRE).

Recent cryo-EM studies have revealed that the morphology of ZIKV E protein shell^6,7^ is very similar to that of the other flaviviruses of known structure, such as the dengue virus^8^. Both the viral glycoprotein shells comprise of 180 copies of E and M proteins that arrange in an icosahedral geometry. While the surface-exposed E proteins are crucial in receptor binding and fusion, the hidden M proteins primarily anchor into the lipid membrane covering the viral RNA. The E protein consists of 504 amino acids with the N-terminal 403 residues forming the ectodomain that folds mainly in β-sheets. Two of these E proteins assemble in the form of head-to-tail homodimers, with three such homodimers lying parallel to each other to form a flat-boat like surface known as raft (Fig. 1). There are 30 such rafts which constitute the whole viral glycoprotein shell. Also, each E protein is comprised of three domains: DI, DII, and DIII with domain DI bridging between domains DII and DIII (Fig. 1C). The intersection of three rafts at the periphery of three DI domain constitutes a 3-fold vertex and five rafts at the periphery of five DIII domains constitutes a 5-fold vertex on the virus surface^6^ (Fig. 1A,B). There are such twenty 3-fold and twelve 5-fold vertices on the ZIKV/DENV glycoprotein shell.

Although the cryo-EM studies have revealed similar structures of the E protein shell of Zika and other flaviviruses^6–10^, the more recent reports based on various biological assays have suggested that ZIKV can sustain the harsh conditions. For example, when exposed to a temperature of 37 °C, Fibriansah *et al*. found significant expansion in the E protein shell of DENV with hole at its all 3-fold vertices^9^. Rossmann and coworkers reported a conformational transition of dengue virus from smooth to bumpy surface due to the change in temperature^10^. Later, Lim *et al*. found serotype-specific expansion in this virus with the DENV serotype 2 (DENV2) exhibiting notable instability in its structure at 37 °C^11^. On the contrary, Kostyuchenko *et al*. have shown that the incubated ZIKV samples at 37 °C have smooth surfaced particles^7^. Also, their plaque assays showed little change in infectivity of ZIKV, whereas the infectivity of DENV2 was greatly reduced with increasing temperature^7^. It is to be noted that the observed differences in infectivity by Kostyuchenko *et al*. were not assessed in human cells, nor *in-vivo* and hence do not provide any direct link between the structural stability and infectivity. Though the increasing number of dengue infections indicates its adaptability to the human body temperature (36.5 to 37.5 °C), several studies have highlighted the effect of temperature on the structure of DENV. However, the molecular basis of this greater stability of ZIKV over DENV2 is unknown. In this study, we attempt to understand the underlying molecular mechanism of the differential stability of ZIKV and DENV2 (NGC strain) at 37 °C.

Even though the cryo-EM studies have provided important information about the structures of different flavivirus E protein shells, the atomistic details pertaining to their differential stability is yet to be known. Here, we employ atomistic molecular dynamics simulations to explore the dynamical changes in virus protein shell structures, subjected to high temperature. We specifically focus on the viral glycoprotein shell, since this constitutes the first level of protection to the viral RNA and thus contributes significantly to the viral stability. Molecular dynamics (MD) simulation is a state-of-the-art computational method that can capture time-dependent conformational changes in biomolecules at varied conditions by calculating inter-atomic forces through solving Newton’s second law. This techniques can not only apprehend the time-dependent changes that the virus protein shell undergoes^12–15^, but also trace the atomic-level contacts and interactions at protein-protein interfaces which are difficult to capture experimentally.

Our simulation results show that while the glycoprotein shell of ZIKV was intact at high temperature, the glycoprotein shell of DENV2 loosened up through the raft-raft interfaces triggered by the formation of holes at 3- and 5-fold vertices. The stronger raft-raft interfaces on ZIKV protein shell showed the presence of multiple polar and H-bonding interactions, in comparison to the weak hydrophobic interactions on DENV2 glycoprotein shell surface. Protein structural network created at the representative vertices validated these findings by exhibiting stronger inter-raft communications in the interlocking FG-loops among five DIII domains in ZIKV.

## Results and Discussion

We performed atomistic MD simulations of ZIKV and DENV2 glycoprotein shells at 37 °C, starting from the available cryo-EM structures of ZIKV (PDBid: 5IRE)^6^ and DENV2 (PDBid: 3J27)^8^. United-atom MD simulation for 40 ns duration was carried out for each of the ZIKV and DENV2 shell at 37 °C, along with the replica simulations of 20 ns for each system (Supplementary Table S1). As a preliminary analysis, deviations in the glycoprotein shell from the starting structures were calculated in terms of the protein backbone RMSD. Results show a slow equilibration, even though the RMSDs reaching to a plateau beyond 30 ns (see Supplementary Fig. S1A, B). Results also suggest the appearance of compact and smooth surfaced ZIKV *versus* loose and rough surfaced DENV2 glycoprotein shell at this increased temperature. The broken DENV2 glycoprotein shell structure matched very well with another cryo-EM structure of dengue reported at 37 °C (PDBid: 3ZKO)^9^. The molecular basis of this temperature sensitivity of DENV2 *versus* insensitivity of ZIKV is discussed below.

### Compact and smooth surfaced ZIKV *versus* loose and rough surfaced DENV2 glycoprotein shell

**Figure 2:**
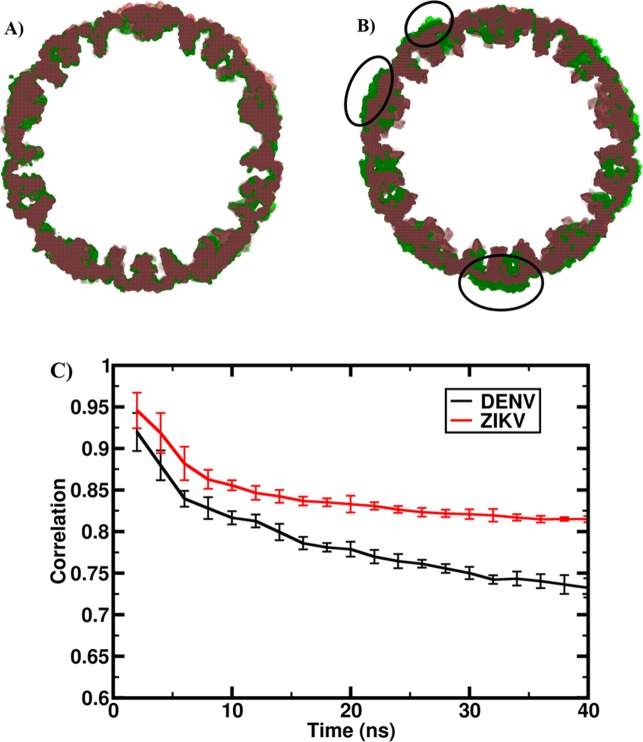
Comparison of the MD-derived and cryo-EM density maps. Results are shown for the aligned density maps of a slice of the glycoprotein shell, cut at the middle. The aligned slices are obtained from MD last frame (green) and starting cryo-EM map (red) of (**A**) ZIKV glycoprotein shell (PDB ID: 5IRE)^6^ and (**B**) DENV glycoprotein shell (PDB ID: 3J27)^8^. The circles highlight the regions of maximum deviation. The density maps from both simulation data and EM coordinates were generated using VMD 1.9.3 software tool (http://www.ks.uiuc.edu/Research/vmd/)^29^. **(C**) Correlation between the simulated and cryo-EM density maps.

In agreement with the reported data of greater temperature insensitivity of ZIKV over DENV2, visual inspection of the simulation trajectories revealed greater stability of the ZIKV glycoprotein protein shell than DENV2 at the simulated temperature of 37 °C. To quantify the observed differences, we first aligned the MD generated density maps with the starting cryo-EM maps and the results are shown in Fig. 2. As evident, the aligned density maps from the MD data and cryo-EM structures show a reasonably good match for ZIKV, while they exhibit large non-overlapping regions for DENV2. The structural correlation with respect to the cryo-EM map was maintained above 0.82 for ZIKV, but it displayed a steady decline to 0.73 for the DENV2. This decline in correlation was also observed in the replica simulations with the values reaching to 0.87 for ZIKV and 0.79 for DENV2 as shown in Supplementary Fig. S1C.

**Figure 3:**
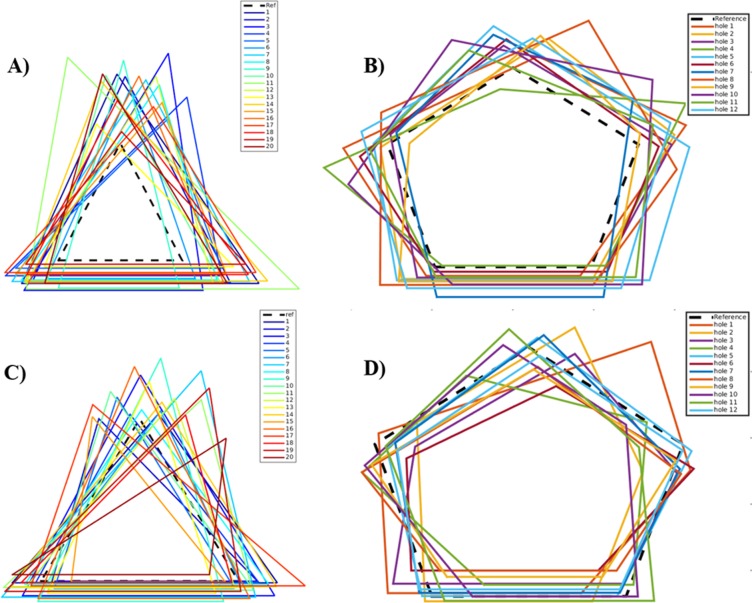
ZIKV 3- and 5-fold vertices remain compact at 37°C. Time-averaged perimeters of all the twenty 3-fold and twelve 5-fold vertices of (**A**,**B**) DENV2 and (**C**,**D**) ZIKV from MD simulations, respectively. The calculated perimeters from EM structures are included in black dotted lines.

Interestingly, the resultant structure of DENV2 from our simulation at elevated temperature matches very well with another cryo-EM structure of DENV2 solved at 37 °C^9^. In close agreement with this EM structure, our simulated protein shell exhibits holes at all 3-fold vertices. The holes are quantified by calculating the time-averaged perimeters of the 3-fold vertices from the simulation data. As shown in Fig. 3 and Supplementary Fig S2, all the twenty 3-fold vertices in DENV2 were greatly distorted and the perimeters increased significantly from the reference values in the starting EM structure. The values of the enlarged vertices were in the range of 42 to 62 Å with an average perimeter of 55 Å, compared to the starting cryo-EM value of 33 Å. On the contrary, ZIKV glycoprotein shell remained stable with the perimeters spanning between 43 and 58 Å relative to the EM value of 45 Å.

Interestingly, our simulations also showed the occurrences of holes at the dengue 5-fold vertices. The 5-fold perimeters increased to 59–67 Å with an average perimeter of 62 Å, compared to the EM value of 50 Å. In fact, our finding of holes also at 5-fold vertices goes parallel to the observed protrusions between the 3- and 5-fold vertices in the solved cryo-EM structure of DENV2 at 37 °C^9^. On the contrary, the ZIKV 5-fold vertices were quite stable and their perimeters ranged only between 52 and 63 Å relative to the EM value of 57 Å. Thus, our results signify smoother ZIKV *versus* rough DENV2 glycoprotein shell surface, in consistent with the experimental data of Kostyuchenko et al^7^. It is also noteworthy from Fig. 3 that among all vertices, DENV2 3-fold vertex 11 (green) underwent the largest deformation. In the subsequent discussion, therefore, we put a special emphasis on the changes experienced by this representative vertex and its constituent rafts.

### Stronger inter- and intra-raft contacts in ZIKV

**Figure 4:**
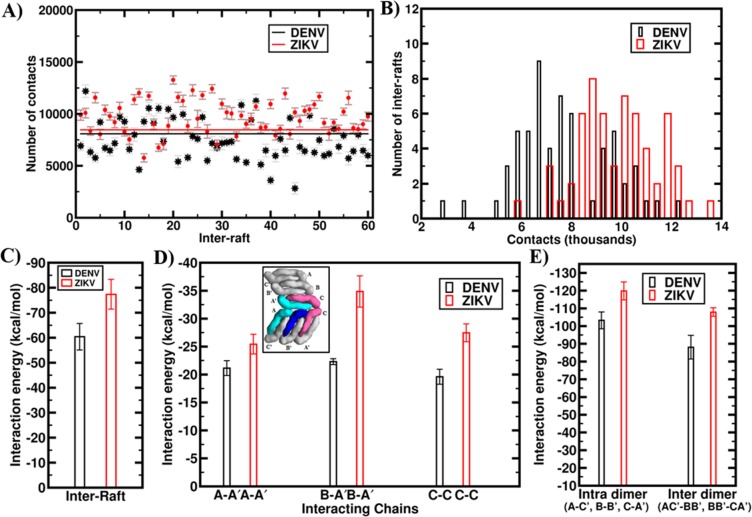
Stronger inter- and intra-raft contacts in ZIKV than DENV2. (**A**) Average number of contacts at all the 60 inter-raft interfaces for ZIKV (red dots) and DENV2 (black stars). Error bars are included. The horizontal lines represent the number of contacts in cryo-EM structures. (**B**) Distribution of inter-raft contacts, calculated with a bin size of 500 contacts for ZIKV (red) and DENV2 (black). (**C**) Average inter-raft interaction energy in ZIKV (red) and DENV2 (black). (**D**) Average interaction energy between two E protein molecules at the ZIKV (red) and DENV2 (black) inter-raft interfaces. Inset shows the 2D distributions and interactions between specific E protein molecules from two neighboring rafts. This 2D image is generated using Chimera 1.11 software suite (http://www.rbvi.ucsf.edu/chimera)^30^. (**E**) Average inter- and intra-dimer interaction energy in ZIKV (red) and DENV2 (black).

Apart from the formation of holes at 3- and 5-fold vertices as seen above, visualization of the MD trajectory also revealed the weakening of inter-raft interfaces in DENV2. To quantify the observed changes, we calculated the number of contacts at all 60 inter-raft interfaces that are present on the whole glycoprotein shell. Here, a contact is considered to form if the atom-atom distance of two residues from the neighbouring rafts falls below 7 Å. Figure 4A shows the number of contacts for all inter-raft interfaces averaged over the final 20 ns simulation data. Results clearly show a significant reduction in the number of contacts in DENV2, while ZIKV retained similar contacts as were present in the starting cryo-EM structure. It is worth mentioning here that the number of contacts in both ZIKV and DENV2 starting cryo-EM structures were very similar (~ 8000), as shown by the horizontal lines in Fig. 4A. We also performed the cluster analysis to see the differences more explicitly. As Fig. 4B shows, the majority of the inter-raft interfaces in DENV2 loose contacts, while the ZIKV shell becomes compact with certain degree of increased contacts.

Along similar line, in Supplementary Fig. S3A, we depicted the two-dimensional representation of a representative 3-fold vertex of ZIKV and DENV2. As evident, the vertex experienced negligible changes in ZIKV, while it weakens in DENV2 with loss of contacts at the raft-raft interfaces. We have calculated the time evolution of the number of contacts at each of the three raft-raft interfaces and shown in Supplementary Fig. S3B. The results suggest that the number of contacts in the constituting interfaces in ZIKV remains fixed or increased, while in DENV2 the contacts reduced significantly. There was a total decrease of ~11000 number of contacts (51.2% decrease) in DENV2, compared to ~ 15% increase in ZIKV contacts. All the three raft-raft interfaces in DENV2 exhibited a reduction of 49%, 68%, and 38% contacts compared to their respective values in the EM structure. A similar reduction in inter-raft contacts was also observed in the adjoining 5-fold vertex of DENV2 (see Supplementary Fig. S4).

To make a direct comparison of the raft-raft interactions in ZIKV *versus* DENV2, we then calculated the average raft-raft interaction energy over the three inter-raft interfaces present at the representative 3-fold vertex shown in Fig. 4A. The energy values were computed over the first-half of simulation data, since the DENV2 glycoprotein shell started exhibiting instability beyond this point. As Fig. 4C shows, the average raft-raft interaction energy was significantly larger in ZIKV (−78.36 kcal/mol) compared to DENV2 (−60.37 kcal/mol). To find the respective contributions of the constituent E protein molecules, we subsequently split the total raft-raft interaction energy into protein-protein interaction energy. As Fig. 4D inset shows, the interactions at a raft-raft interface is primarily contributed by the association of constituting protein molecule A from one raft with protein molecule A’ of the adjacent raft (A-A’), association of protein molecule B of one raft with protein molecule A’ of the adjacent raft (B-A’), and association between protein molecule C of the first raft with protein molecule C of the second raft (C–C). Figure 4D shows the comparison of these protein-protein interactions at ZIKV and DENV2 inter-raft interfaces. It is evident that protein-protein interactions in ZIKV is much greater than DENV2 with an energy difference of ~3 kcal/mol at A-A’ interface, ~11 kcal/mol at B-A’ interface, and ~7 kcal/mol at C-C interface.

We also looked into the intra-raft association by calculating the inter-dimer AC’-BB’, BB’-CA’ and intra-dimer A-C’, B-B’, C-A’ interaction energies (see Fig. 4D inset for the protein molecule nomenclature). Results in Fig. 4E suggest that intra-raft interactions - both inter-dimer and intra-dimer – are not too different in ZIKV and DENV2. However, the noteworthy features that emerge from comparing Fig. 4D,E are - (i) inter-raft interfaces are always weaker than the intra-raft interfaces, (ii) intra-dimer association is the strongest among all the protein-protein interactions present, in both ZIKV and DENV2. These results are consistent with the experimental report showing that E proteins exist as dimers in solution^16^. Overall, our results suggest that ZIKV has stronger intra- and inter-raft interactions that make this virus glycoprotein shell stronger than the DENV2.

### Electrostatics and H-bonding interactions impart greater ZIKV inter-raft stability

From the above results it is evident that DENV2 glycoprotein shell is more susceptible to break at high temperature through its inter-raft interfaces. To find the constituent E protein residues that were responsible for such differential inter-raft stability of ZIKV *versus* DENV2, we looked into the residue-level energy contribution at the inter-raft interfaces of both the glycoprotein shells. As can be noted from Fig. 4D inset, the stability of A-A’ interface is primarily contributed by the interactions of domain DIII residues of A molecule with the domain DI residues of A’ molecule of the second raft (see Fig. 1 for domain names). Similarly, at B-A’ interface, it is the interaction of DIII domain residues of B molecule with the DII domain residues of A’ molecule of the second raft that were responsible for the stability. The C-C interface involves the interactions of DIII domain residues of C molecules from the adjacent rafts.

**Figure 5:**
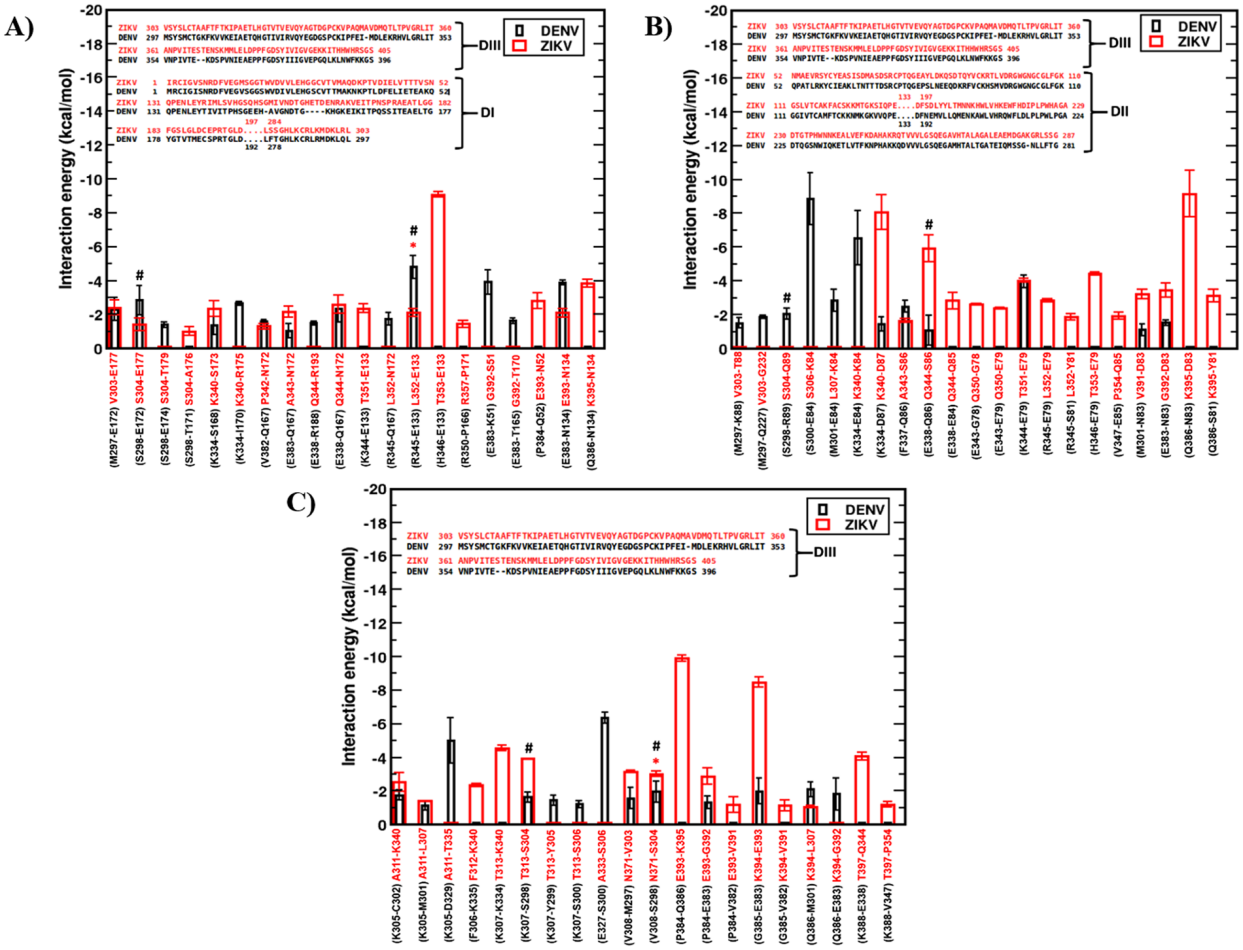
Residue-level energy contributions. Energy contributions at the three protein-protein interfaces - (**A**) A-A’, (**B**) B-A’, and (**C**) C-C in ZIKV (red) and DENV2 (black) with domain-wise sequence alignments of two E proteins depicted in insets. See Fig. 4D inset for E protein notations.

Figure 5(A–C) show the residue-level energy contributions at the three inter-raft regions - A-A’, B-A’, and C-C in both ZIKV and DENV2 with the domain-wise sequence alignments of two viral E proteins depicted in the insets. Results are shown for the residue-pairs that contributed ≥ −1 kcal/mol to the total energy of the respective inter-raft interfaces. As Fig. 5A shows, at the inter-raft region A-A’, nearly equal number of residue pairs contribute to the total energy in ZIKV and DENV2. However, while majority of these residue pairs were involved in electrostatic and H-bonding interactions in ZIKV, they involve in hydrophobic or weakly electrostatic interactions in DENV2. For example, the residue pairs Thr353_r1_-Glu133_r2_, Lys395_r1_-Asn134_r2_, Gln344_r1_-Asn172_r2_ from two neighbouring rafts, r1 and r2 in ZIKV showed > −4 kcal/mol energy contribution, in contrast to the very little contribution of the corresponding residue pairs in DENV2. Though the residues Glu133_r2_ and Asn134_r2_ were conserved in domain I of both viral proteins, domain III showed substitution from Thr353_r1_ and Lys395_r1_ in ZIKV to His346_r1_ and Gln386_r1_ in DENV2. While the ZIKV Thr353_r1_ could involve in strong sidechain-sidechain H-bonding interactions with Glu133_r2_ and Lys395_r1_ could interact electrostatically with Asn134_r2_; DENV2 substitutions fail to sustain such favourable interactions. Similarly, stable H-bond was observed between the side chain of Gln344_r1_ and backbone amide of Asn172_r2_ in ZIKV. H-bond lifetime analysis have shown that both the above H-bonds persisted up to 70% of simulation time (see Supplementary Fig. S5). On the contrary, no H-bond existed more than 20% of simulation time in DENV2. These results also match favourably well with the ZIKV and DENV2 cryo-EM structure data. For instance, the observed H-bond between backbone amide of Leu352 and sidechain of Glu133 in ZIKV cryo-EM structure^6^ was persistent over 50% of the simulation time (indicated by * in Figs. S5, 5A). On the contrary, the reported H-bonds between Ser298 and Glu172 and between Arg345 and Glu133 in the DENV2 cryo-EM structure^8^, existed only for 12% and <10% of the simulation time (indicated by # in Figs. S5, 5A), suggesting significantly weaker interactions in DENV2, which further weakens at higher temperature. It is worth mentioning here that some of the aforementioned H-bonds in the cryo-EM structures were observed upon modelling the missing atoms/residues.

In consistence with the large inter-raft energy difference at B-A’ interface (see Fig. 4D), the number of ZIKV residue pairs contributing to this interface energy is found to be significantly larger than DENV2 as shown in Fig. 5B. Among these, the residue pairs Lys340_r1_-Asp87_r2_, Thr353_r1_-Glu79_r2_, and Lys395_r1_-Asp83_r2_ in ZIKV showed large contribution, in contrary to the negligible interaction exhibited by the corresponding residue pairs Lys334_r1_-Asp87_r2_, His346_r1_-Glu79_r2_, and Glu386_r1_-Asn83_r2_ in DENV2. While the sidechain-sidechain H-bond between Thr353_r1_ and Glu79_r2_ was very stable and persistent in ZIKV (see Supplementary Fig. S6 for % H-bond persistence), the substituted His346_r1_ in DENV2 showed negligible interaction with Glu79_r2_. Similarly, the polar Lys395_r1_-Asp83_r2_ interaction in ZIKV was completely absent in DENV2 due to the sidechain-sidechain repulsion of substituted Glu386_r1_ with Asn83_r2_. Nonetheless, a few favorable interactions were present between the DENV2 residue pairs Ser300_r1_-Glu84_r2_, Lys334_r1_-Glu84_r2_, Met301_r1_-Glu84_r2_ etc. When compared with the EM data, the H bonds formed by Ser298_r1_ and Glu338_r1_ respectively with Arg89_r2_ and Gln86_r2_ in DENV2 cryo-EM structure were retained only for 10% of simulation time (indicated by # in Figs. S6, 5B). On the other hand, the cryo-EM structure H-bonds for ZIKV residue pairs Leu352 and Glu79, Thr353 and Glu79, Gly392 and Asp83 persisted>50% of the simulation time (see Supplementary Fig. S6).

At C-C interface of ZIKV, the inter-raft stability was facilitated majorly by the residue pairs Glu393_r1_-Lys395_r2_ and Lys394_r1_-Glu393_r2_ (Fig. 5C). These residues involve in strong electrostatic interactions and contribute significantly toward the total inter-raft stabilization energy. In DENV2, this sequence of charged residues EKK is substituted by PGQ, which fails to stabilize the 5-fold vertex effectively, making it susceptible to temperature. Apart from these strong electrostatic interactions, the C-C interface in ZIKV is also stabilized by multiple H-bond interactions, in contrast to the weak van der Waals interactions in DENV2. H-bond analysis at this interface shows persistent hydrogen bonds in ZIKV that match favorably well with the EM structure data. For example, H-bonds found between Asn371_r1_ and Ser304_r2_, Thr313_r1_ and Ser304_r2_ in ZIKV cryo-EM structure were persistent for>60% of the simulation time (indicated by * in Supplementary Fig. S7). The cryo-EM H-bond between Val308_r1_ and Ser298_r2_ in DENV2 was also stable during the simulations.

Interestingly, a recent structural study had proposed potential H-bond between Gln350_r1_ and Thr351_r2_ in CD-loops of the neighboring rafts as a potential contributor in ZIKV stability^7^. However, our data revealed <2% persistence of this H-bond during the entire simulation period. Instead, our data suggests that the stability of ZIKV raft-raft interfaces stems from electrostatic interactions among the FG-loops (due to EKK residue sequence at positions 393–395) and interactions between Thr353_CD-loop_ and Glu133_e-Eo loop_; Lys340_B-C loop_ and Asp87_b-c loop_; Gln344_C-strand_ and Ser86_b-c loop_; Lys395_F-G loop_ and Asp83_b-c loop_. Thus, from the interaction energy data in Fig. 5, we have identified the inter-raft residues Asp83, Asp87, Ser86, Glu133, Lys340, Gln344, Thr353, Glu393, Lys394, Lys395 in ZIKV that play key roles in the ZIKV glycoprotein shell stability.

### Protein structural network reveals stronger inter-raft communications in ZIKV

**Figure 6:**
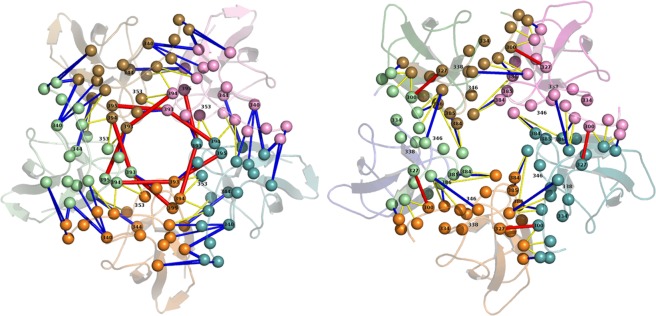
Residue level energy network. Protein structural network at a 5-fold vertex of ZIKV (above) and DENV2 (below). For clarity, five constituting E protein are colored differently. Color code for interactions - red: electrostatic, blue: H-bond, yellow: vdW.

A protein structural network often helps visualizing the complex interactions in protein-protein conjugates in a more tractable representation. Hence, we generated the structural network of a representative 5-fold vertex of ZIKV and DENV2 by combining the (significant) interactions at five adjoining C-C interfaces (i.e. five DIII domains at a 5-fold vertex). A protein structural network consists of nodes represented by aminoacids, connected to each other by edges that can be represented by different parameters. Studies have shown that the use of interaction energies as edge weights between nodes can efficiently capture the structural stability of protein-protein complexes^17^. Hence, we represent the ZIKV and DENV2 5-fold vertices in the form of a network based on the pairwise interaction energies of their constituent aminoacids, and the results are shown in Fig. 6. In the Figure, the Cα atoms of the interacting nodes are connected by edges representing their interactions, while the edge thicknesses represent the strength of interaction and color code represents the nature of interaction (red: electrostatic interactions > −5 kcal/mol, blue: H-bond interactions −2 to −5 kcal/mol, yellow: vdW interactions < −2 kcal/mol). It is evident from Fig. 6 that inter-raft communications in ZIKV are more robust through multiple electrostatic and H-bond interactions. Particularly, the intricate network of interlocking FG-loops stabilized by residues Glu393, Lys394, Lys395 was significantly stronger that makes this protein shell stable, even at high temperature or harsh conditions. On the other hand, the weaker communications in DENV2, majorly assisted by feeble vdW interactions and few H-bonds, make this protein shell prone to break. A similar difference in the 3-fold structural network is also observed as shown in Supplementary Fig. S8. However, in accordance with the cryo-EM data at 37 °C that 3-fold vertices are more prone to break, the weaker 3-fold vertex showed lesser inter-raft communications than the 5-fold vertex. Nevertheless, stronger *versus* weaker communications in ZIKV *versus* DENV were evident.

We understand that in reality both viral glycoproteins (E and M) are embedded in a lipid bilayer, which would have a significant impact on the movement of the glycoproteins during temperature-induced fluctuations. Here we have omitted the bilayer for simplicity. We believe that the presence of bilayer would have impacted our results quantitatively. For example, the strong interactions between the lipid bilayer and M protein shell would have pushed our simulations to a longer time-scale to capture the changes at the same extent as observed here. Moreover, if the simulations were run at the melting temperature of the bilayer, the observed protrusions could have been more pronounced! However, all these explorations would require at least coarse-graining of the systems.

## Summary and Conclusions

In summary, our results suggest a stronger inter-raft communications on ZIKV E protein shell surface through multiple electrostatic and H-bond interactions that makes this virus less temperature-sensitive. We propose that the identified ZIKV residues could be potential targets for designing small molecule inhibitors to inhibit E protein assembly and membrane fusion, similar to the recently reported HIV and DENV2 envelope-targeted inhibitors^18–20^. It’s worth mentioning here that recently a set of monoclonal antibodies against various flaviviruses, including ZIKV has been tested^21–24^. These virus-neutralizing antibodies were primarily found to target the DIII domain of viral E protein. It was encouraging to see that six of the ten inter-raft residues that we found crucial for ZIKV stability (Lys340, Gln344, Thr353, Glu393, Lys394, Lys395) belonged to the DIII domain. Since there are several other factors involved in the development of antibodies, such as the immunogenicity of the given region on the protein, we ran a prediction of antigenic determinants by Kolaskar and Tongaonkar algorithm to check if our identified residues are good targets for Ab development^25^. Out of the six, three residues - Lys340, Gln344, Thr353 were found to fall in the antigenic peptide sequence (see Supplementary Table S2). Based on our detailed residue-level information, specific ZIKV (and DENV2) antibodies and small molecule inhibitors can be designed. We hope that our findings will motivate experimental designs to combat the spread of the flaviviruses.

## Methods

### System preparation

Cryo-EM structures of the glycoprotein shell of ZIKV (PDBid: 5IRE)^6^ and DENV2 (strain NGC) (PDBid: 3J27)^8^ solved at 3.8 Å and 3.5 Å resolution were used to prepare the initial conformations for simulations. The missing atoms and ZIKV E protein missing residues, 502:V-S-A:504 were incorporated using modeller 9v13 tool^26^. Similarly, the DENV2 M protein missing residues, 73:S-M-T:75 were included. The prepared systems were subjected to united-atom (UA) simulations to explore their structural stability and inherent dynamics.

### Simulation details

We carried out 40 ns long united-atom MD simulation for each of the ZIKV and DENV2 glycoprotein shells along with the 20 ns long replica simulations by changing the initial velocities (see Supplementary Table S1). It is worth mentioning here that simulating these large systems of ~ 12 million atoms required extensive computational time, and the runtime for 1 ns simulation in our available resource of 256 cores Intel Xeon E5-2650 processors varied from 48–52 hrs. Hence, simulating these systems for more than 40 ns was difficult. Nevertheless, as shown above, this time length was sufficient to extract new insights, since the results converged to the reported cryo-EM data.

The two viral glycoprotein shells were subjected to UA simulations at 37 °C using GROMOS96 53A6 forcefield^27^. In the UA simulations, except for the non-polar hydrogens, each other atoms of the virus glycoprotein shells were described explicitly. The non-polar hydrogens were embedded with the heavy atoms to which they were bonded. Initially, the systems were briefly minimized using steepest descent and conjugate gradient algorithms, followed by solvation with explicit water (SPC model) in cubic periodic box. The interior of the glycoprotein shells was thoroughly packed with water. The salt concentration of 0.15 M was maintained. The solvated systems were then subjected to extensive energy minimization, followed by thorough equilibration in NPT ensemble to adjust the solvent density. A cut off of 1.0 nm for both van der Waals (vdW) and electrostatic interactions and particle mesh Ewald sum with real space cut-off at 1.0 nm were used. LINCS algorithm was used to constrain all bonds involving hydrogen atoms. The systems were equilibrated for 1 ns with a time step of 2 fs. Finally the production run was performed for 40 ns for each ZIKV and DENV2 system. The above simulation protocol was followed for the 20 ns long replica simulations also. All the simulations were performed using Gromacs  2018 simulation software^28^. We utilized gromacs trajectory analysis tools and in-house scripts to extract the information from simulation data. The density maps from simulation data were generated using the volmap tool in VMD^29^ at a resolution of 1.0 Å. Chimera^30^ was used to visualize and align the density maps to compute the correlation. Molecular mechanics generalized Born surface area (MMGBSA) method is used to calculate the inter- and intra-raft interaction energies, and subsequently residue-level decomposition was performed to obtain residue-residue interactions^31^.

## Supplementary information

Supplementary Information.

## References

[1] Baud D, Gubler DJ, Schaub B, Lanteri MC, Musso D (2017). An update on Zika virus infection. The Lancet.

[2] Pierson TC, Diamond MS (2018). The emergence of Zika virus and its new clinical syndromes. Nature.

[3] Foy BD (2011). Probable non-vector-borne transmission of Zika virus, Colorado, USA. Emerg. Infect. Dis..

[4] Musso D (2015). Potential Sexual Transmission of Zika Virus. Emerg. Infect. Dis..

[5] Lindenbach, B. D., Thiel, H.-J. & Rice, C. M. *33 Flaviviridae: The Viruses and Their Replication*. (2006).

[6] Sirohi D (2016). The 3.8 Å resolution cryo-EM structure of Zika virus. Science.

[7] Kostyuchenko VA (2016). Structure of the thermally stable Zika virus. Nature.

[8] Zhang X (2013). Cryo-EM structure of the mature dengue virus at 3.5-Å resolution. Nat. Struct. Mol. Biol..

[9] Fibriansah G (2013). Structural Changes in Dengue Virus When Exposed to a Temperature of 37 C. J. Virol..

[10] Zhang X (2013). Dengue structure differs at the temperatures of its human and mosquito hosts. Proc. Natl. Acad. Sci. USA.

[11] Lim X-X (2017). Conformational changes in intact dengue virus reveal serotype-specific expansion. Nat. Commun..

[12] Perilla JR, Hadden JA, Goh BC, Mayne CG, Schulten K (2016). All-Atom Molecular Dynamics of Virus Capsids as Drug Targets. J. Phys. Chem. Lett..

[13] Perilla JR, Schulten K (2017). Physical properties of the HIV-1 capsid from all-atom molecular dynamics simulations. Nat. Commun..

[14] Reddy T, Sansom MSP (2016). The Role of the Membrane in the Structure and Biophysical Robustness of the Dengue Virion Envelope. Structure.

[15] Marzinek JK, Holdbrook DA, Huber RG, Verma C, Bond PJ (2016). Pushing the Envelope: Dengue Viral Membrane Coaxed into Shape by Molecular Simulations. Structure.

[16] Modis Y, Ogata S, Clements D, Harrison SC (2005). Variable Surface Epitopes in the Crystal Structure of Dengue Virus Type 3 Envelope Glycoprotein. J. Virol..

[17] Appadurai R, Senapati S (2016). Dynamical Network of HIV-1 Protease Mutants Reveals the Mechanism of Drug Resistance and Unhindered Activity. Biochemistry.

[18] Lin P-F (2003). A small molecule HIV-1 inhibitor that targets the HIV-1 envelope and inhibits CD4 receptor binding. Proc. Natl. Acad. Sci..

[19] Schmidt AG, Lee K, Yang PL, Harrison SC (2012). Small-Molecule Inhibitors of Dengue-Virus Entry. PLoS Pathog..

[20] Modis Y, Ogata S, Clements D, Harrison SC (2003). A ligand-binding pocket in the dengue virus envelope glycoprotein. Proc. Natl. Acad. Sci. USA.

[21] Crill WD, Roehrig JT (2001). Monoclonal antibodies that bind to domain III of dengue virus E glycoprotein are the most efficient blockers of virus adsorption to Vero cells. J. Virol..

[22] Beasley DWC, Barrett ADT (2002). Identification of neutralizing epitopes within structural domain III of the West Nile virus envelope protein. J. Virol..

[23] Zhao H (2016). Structural Basis of Zika Virus-Specific Antibody Protection. Cell.

[24] Yang M, Dent M, Lai H, Sun H, Chen Q (2017). Immunization of Zika virus envelope protein domain III induces specific and neutralizing immune responses against Zika virus. Vaccine.

[25] Vita R (2018). The Immune Epitope Database (IEDB): 2018 update. Nucleic Acids Res..

[26] Šali A, Blundell TL (1993). Comparative Protein Modelling by Satisfaction of Spatial Restraints. J. Mol. Biol..

[27] Oostenbrink C, Villa A, Mark AE, Van Gunsteren WF (2004). A biomolecular force field based on the free enthalpy of hydration and solvation: The GROMOS force-field parameter sets 53A5 and 53A6. J. Comput. Chem..

[28] Abraham MJ (2015). GROMACS: High performance molecular simulations through multi-level parallelism from laptops to supercomputers. SoftwareX.

[29] Humphrey W, Dalke A, Schulten K (1996). VMD: visual molecular dynamics. J. Mol. Graph..

[30] Pettersen EF (2004). UCSF Chimera?A visualization system for exploratory research and analysis. J. Comput. Chem..

[31] Mongan J, Simmerling C, McCammon JA, Case DA, Onufriev A (2007). Generalized Born Model with a Simple, Robust Molecular Volume Correction. J. Chem. Theory Comput..

