# Supplementary material for: Molecular Basis of Differential Stability and Temperature Sensitivity of ZIKA versus Dengue Virus Protein Shells
Source: Sci Rep. 2020 May 21;10:8411. doi: 10.1038/s41598-020-65288-3 (PMC7242387; doi:10.1038/s41598-020-65288-3)
Supplement: Supplementary file 1 — Supplementary Information. [file 41598_2020_65288_MOESM1_ESM.pdf]

Supporting Information for

Molecular Basis of Differential Stability and Temperature Sensitivity of Zika *versus* Dengue Virus Protein Shells

Chinmai Pindi,[#] Venkat R Chirasani,[#] Mohd. Homaidur Rahman,[#] Mohd. Ahsan,[#] Prasanna D Revanasiddappa, and Sanjib Senapati*

Department of Biotechnology and BJM School of Biosciences,
Indian Institute of Technology Madras, Chennai 600036, India.
Ph:+91-44-22574122, e-mail: sanjibs@iitm.ac.in

Table S1: List of systems studied in this work.

**Name**	**Simulated System**	**Temperature (°C)**	**No. of atoms**	**Simulation box volume (Å^3)**	**Run time**
ZIKV	ZIKV glycoprotein shell	37	12,157,404	504 X 504 X 504	40ns
DENV	DENV2 glycoprotein shell	37	11,523,800	495 X 495 X 495	40ns
ZIKV	ZIKV glycoprotein shell	37	12,517,713	510 X 510 X 510	20ns
DENV	DENV2 glycoprotein shell	37	11,886,009	497 X 497 X 497	20ns

A)

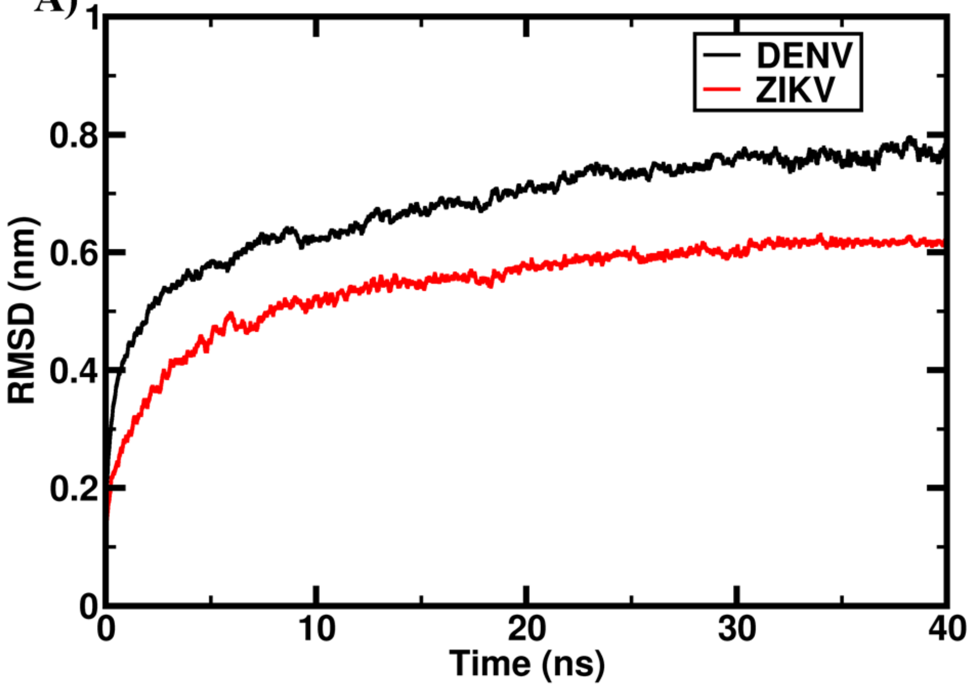
DENV
ZIKV
RMSD (nm)
Time (ns)
0
0.2
0.4
0.6
0.8
1
0
10
20
30
40

B)

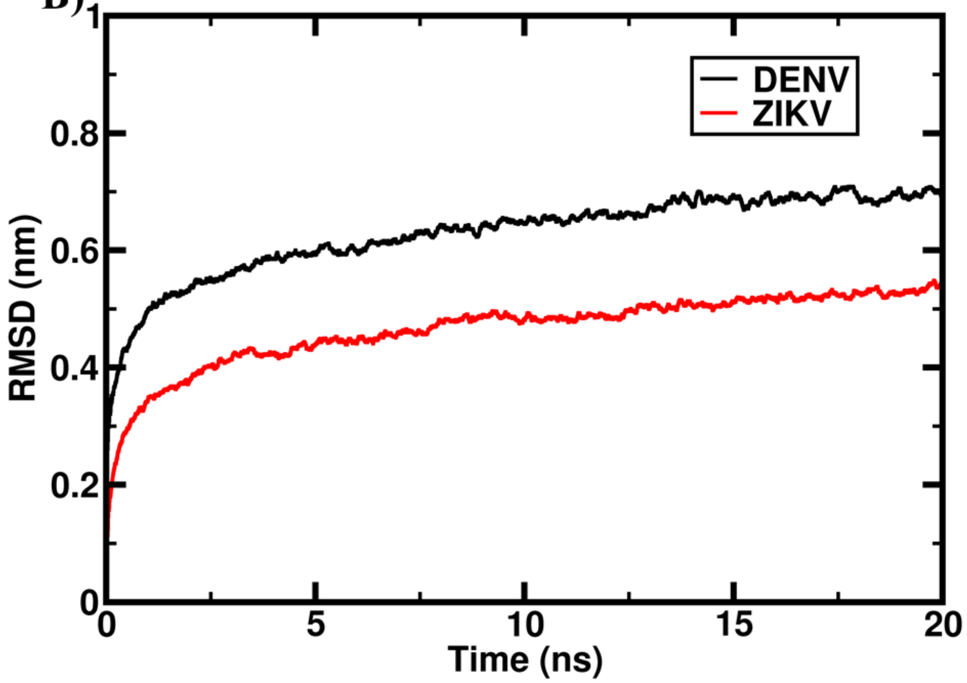
DENV
ZIKV
RMSD (nm)
Time (ns)
0
0.2
0.4
0.6
0.8
1
0
5
10
15
20

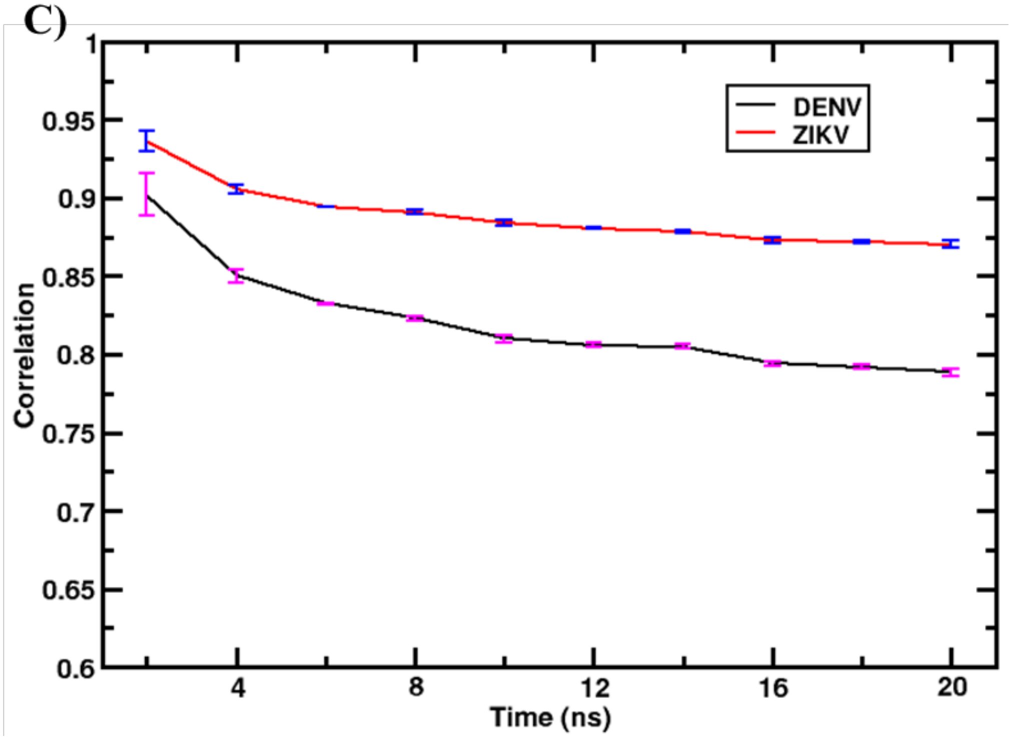

Fig S1: Time evolution of the backbone RMSD of viral glycoprotein shells in the (A) primary simulations (B) replica simulations. (C) Correlation between the simulated and cryo-EM density maps calculated for the replica simulations.

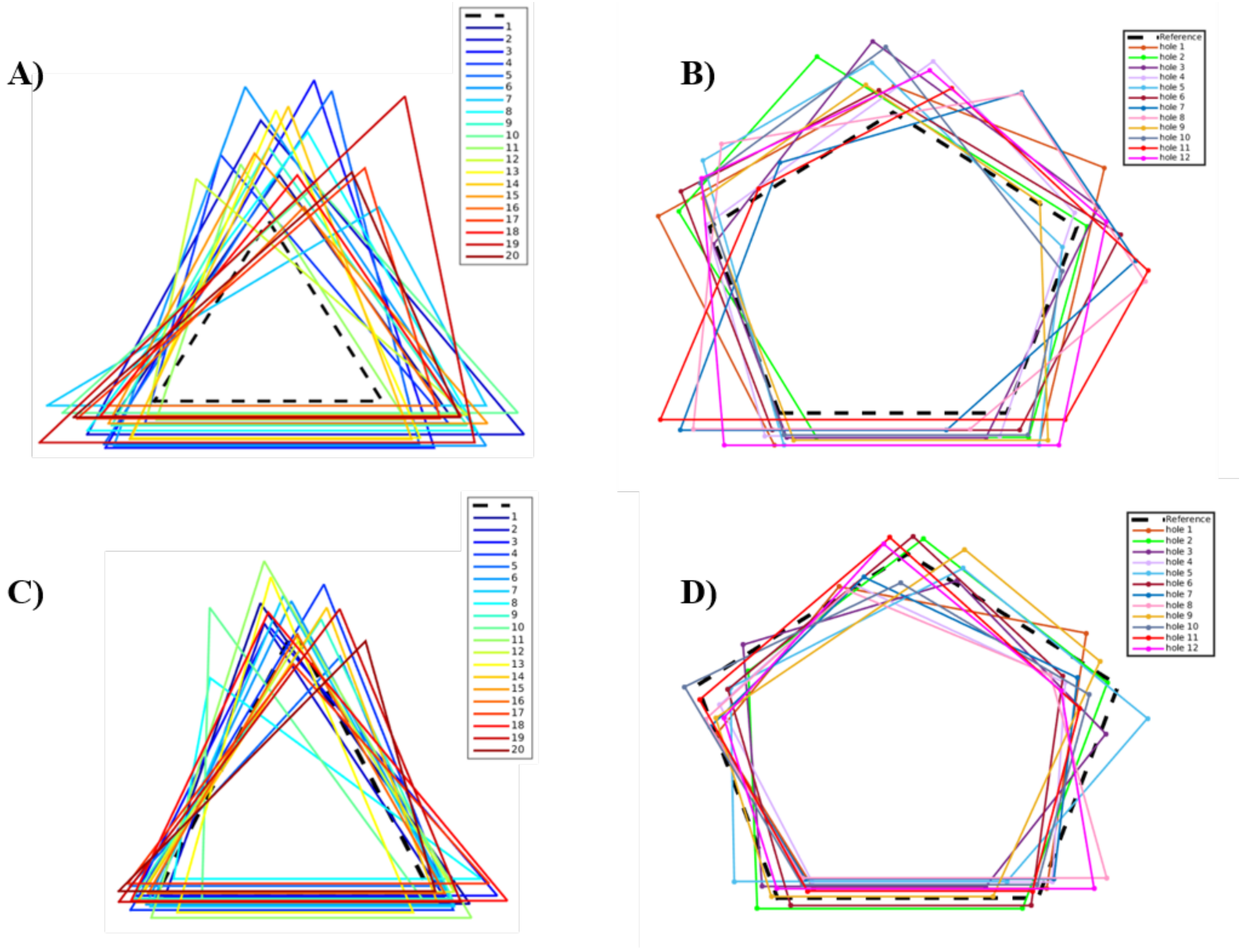

Fig S2: Time-averaged perimeters of all the twenty 3-fold and twelve 5-fold vertices of (A) – (B) DENV and (C) – (D) ZIKV from replica MD simulations, respectively. The calculated perimeters from EM structures are included in black dotted lines. The perimeter of the 3-fold vertices in DENV were in the range of 42 to 57 Å with an average perimeter of 50 Å relative to the EM value of 33 Å while, in ZIKV the perimeters spanned between 44 and 56 Å relative to the EM value of 45 Å. The 5-fold perimeters in DENV increased to 61 - 68 Å with an average perimeter of 65 Å, compared to the EM value of 50 Å. On the contrary, the ZIKV 5-fold vertices were quite stable and their perimeters ranged only between 52 and 62 Å relative to the EM value of 57 Å.

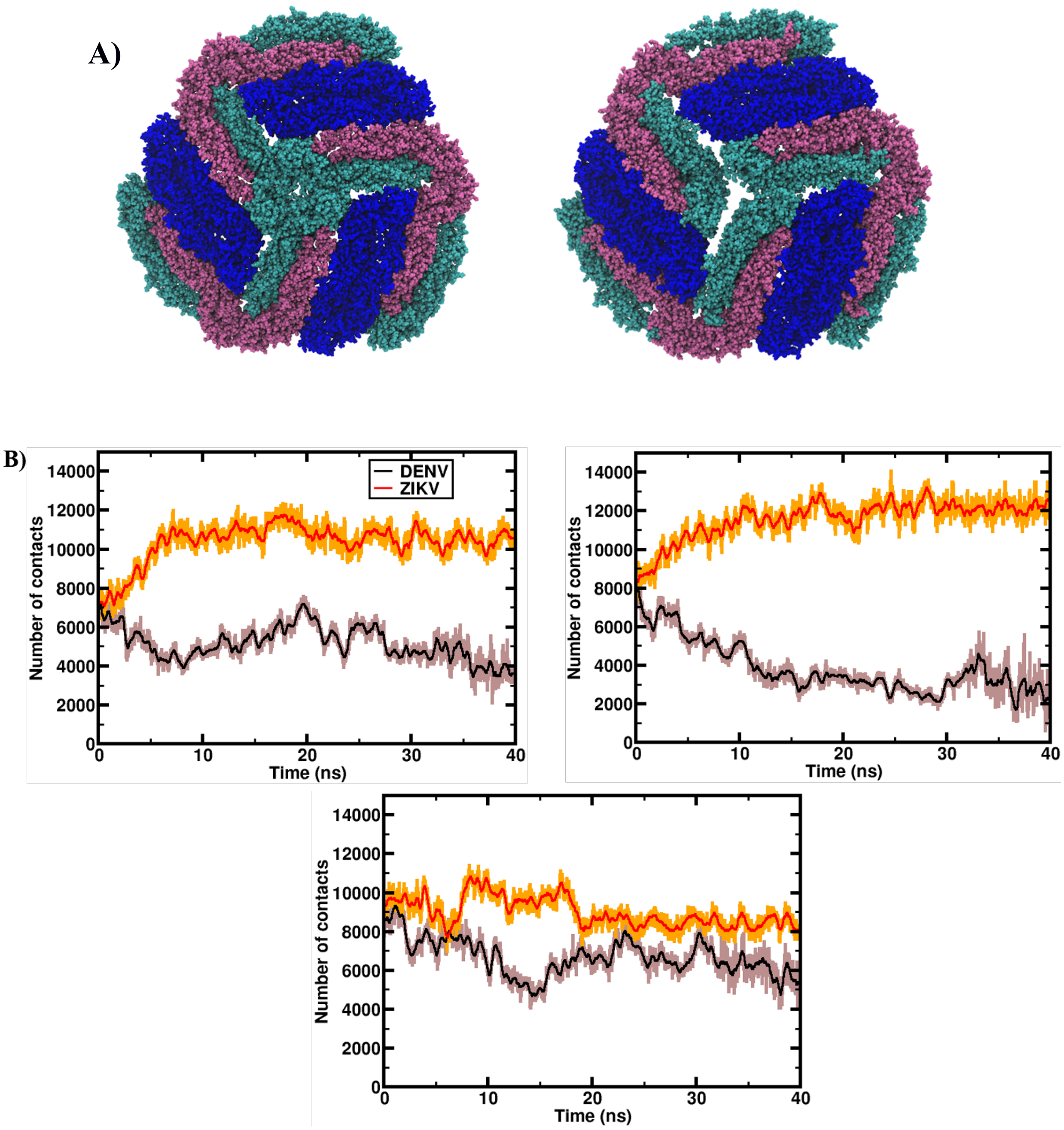

Fig S3: (A) Representative 3-fold vertex in ZIKV and DENV2 from MD simulations at 37°C. Color scheme is similar to Fig. 1A. Figures are generated by VMD 1.9.3 software suite (http://www.ks.uiuc.edu/Research/vmd/)[29]. (B) Comparison of the number of contacts at three inter-raft interfaces present in the 3-fold vertex shown in (A). While the shaded lines represents the contacts calculated for every 20ps frames of the trajectory, the solid line represents the running average along the simulation time scale.

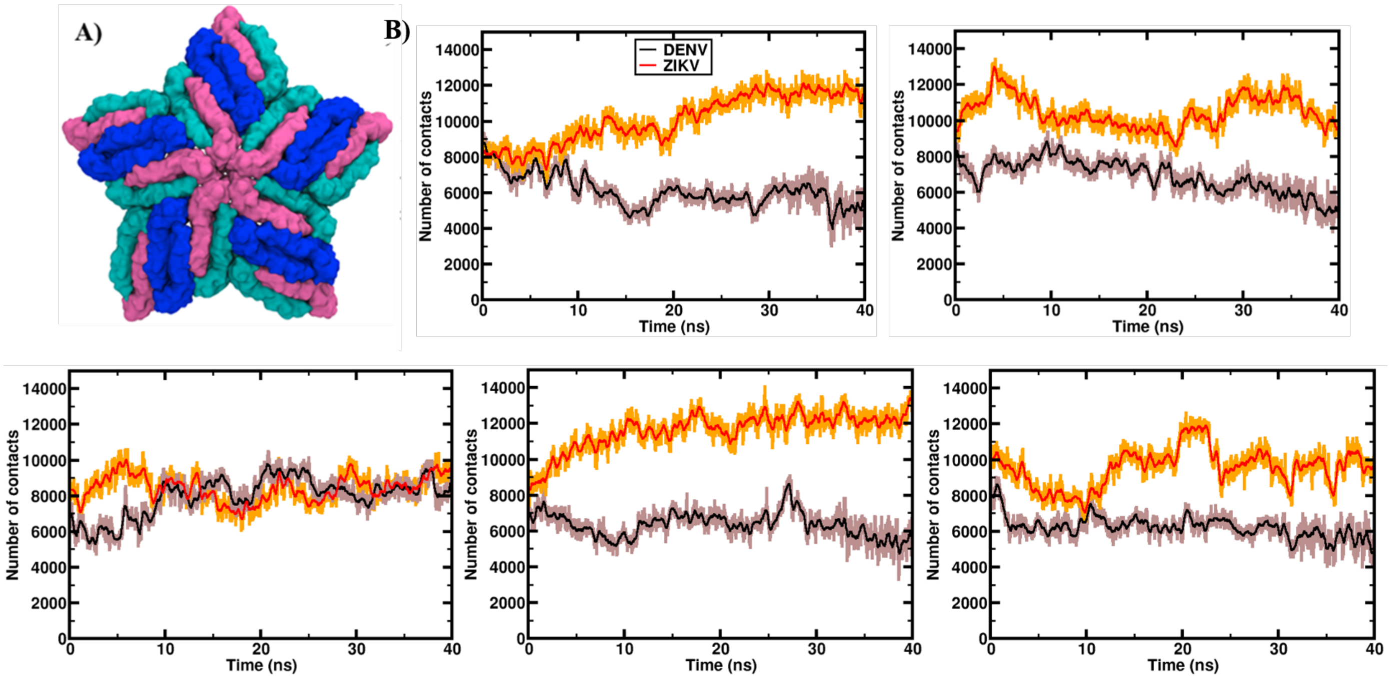

Fig S4: (A) Representative 5-fold vertex in ZIKV from MD simulation at 37^{0}C. The figure is generated by VMD 1.9.3 software suite (http://www.ks.uiuc.edu/Research/vmd/)[29]. Color scheme is similar to Fig. 1A. (B) Comparison of the number of contacts at five inter-raft interfaces present in the 5-fold vertex shown in (A). While the shaded lines represents the contacts calculated for every 20ps frames of the trajectory, the solid line represents the running average along the simulation time scale. The increased contact is due to the breaking of the opposite interface that brings the adjoining rafts somewhat closer.

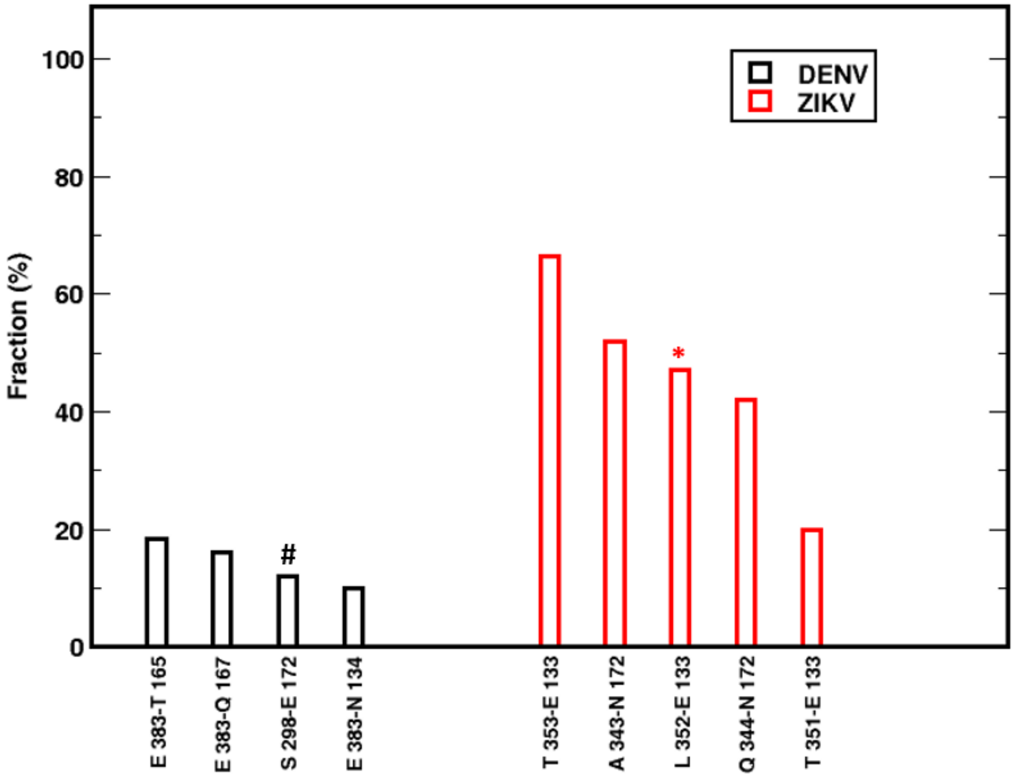

Fig S5: Persistence of H-bond (in %) of the designated residue pairs in ZIKV (red) and DENV (black) at the A-A' interface during the entire simulation run. Only those H-bonds that persisted > 10% of the simulation time are shown. Here, asterisk (*) and hash (#) specify the particular H-bonds that were present in cryo-EM structures of ZIKV and DENV, respectively.

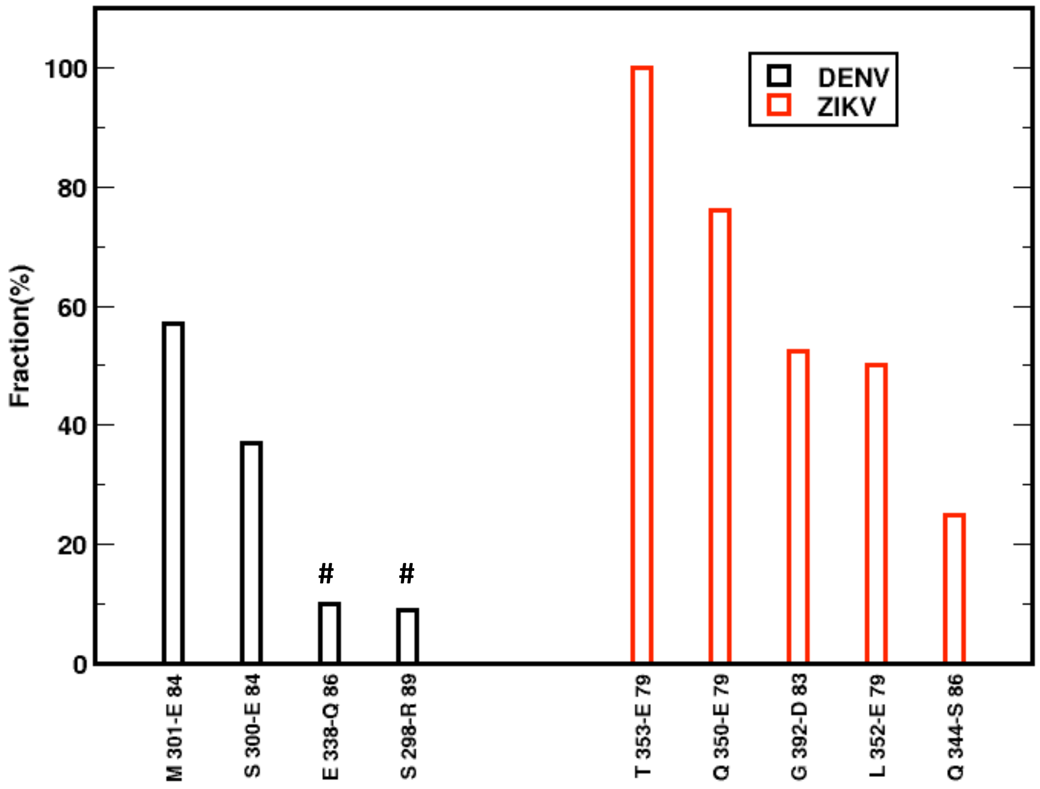

Fig. S6: Percent H-bond lifetime of the residue pairs in B-A' interface. When compared with the EM data, the H bonds formed by $Ser298_{r1}$ and $Glu338_{r1}$ respectively with $Arg89_{r2}$ and $Gln86_{r2}$ in DENV2 cryo-EM structure were retained only for 10% of simulation time (indicated by #). On the other hand, the cryo-EM structure H-bonds for ZIKV residue pairs Leu352 and Glu79, Thr353 and Glu79, Gly392 and Asp83 persisted >50% of the simulation time.

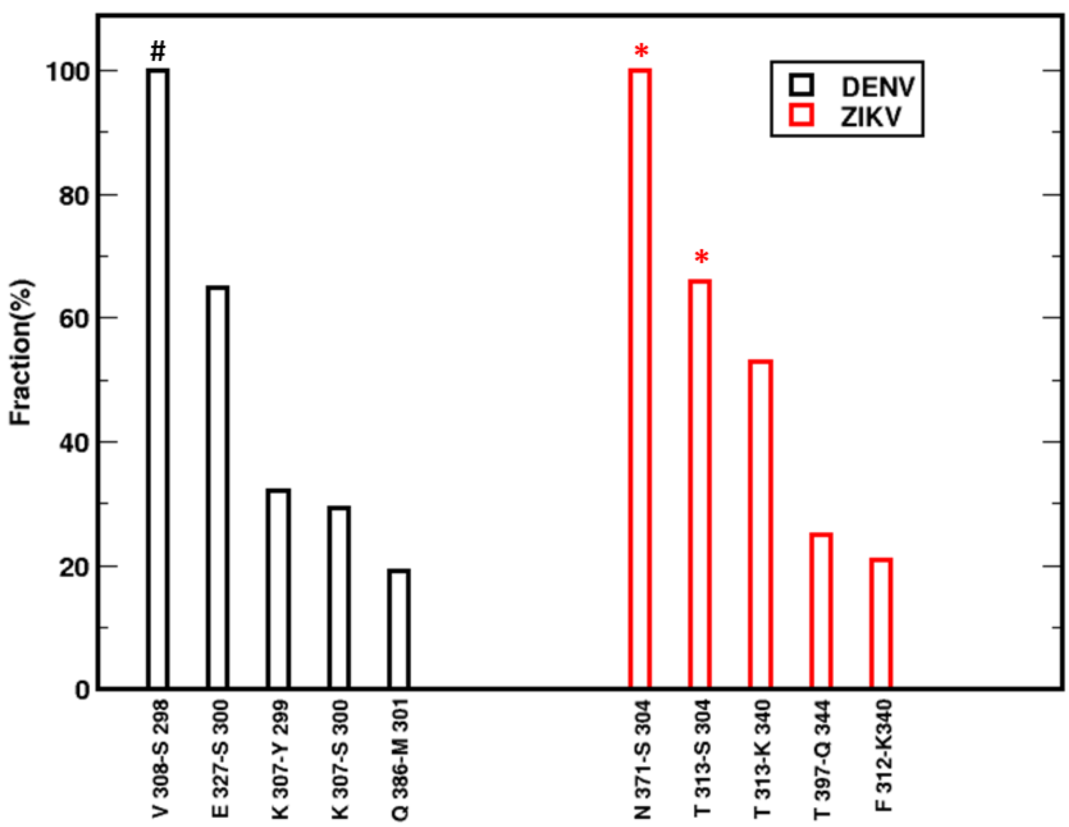

Fig S7: Percent H-bond lifetime of the residue pairs in C-C interface. H-bond analysis at this interface shows persistent hydrogen bonds in ZIKV that match favorably well with the EM structure data. For example, H-bonds found between Asn371$_{r1}$ and Ser304$_{r2}$, Thr313$_{r1}$ and Ser304$_{r2}$ in ZIKV cryo-EM structure were persistent for >60% of the simulation time (indicated by *). The cryo-EM H-bond between Val308$_{r1}$ and Ser298$_{r2}$ in DENV was also stable during the simulations.

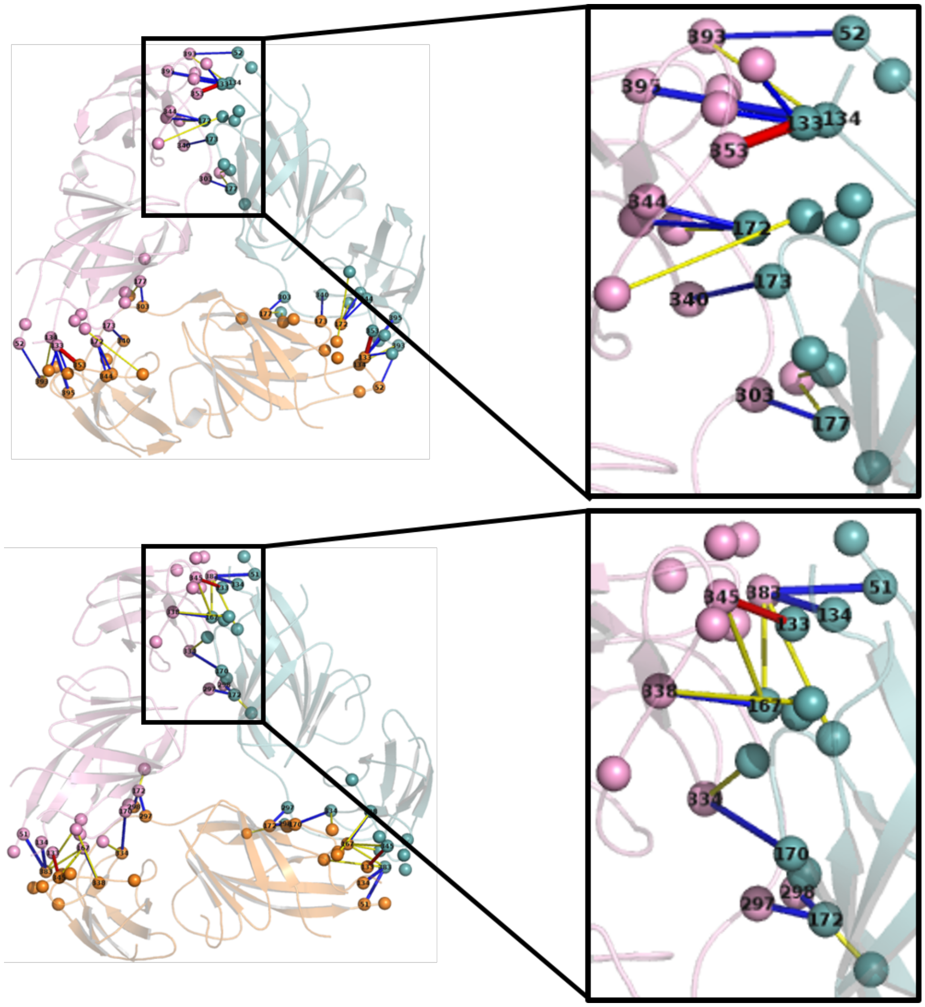

Fig S8: Protein structural network at the representative 3-fold vertices of ZIKV and DENV. For clarity, the three constituting E proteins are colored differently. Color code for interactions - red: electrostatic, blue: H-bond, yellow: vdW. The figure is generated from MD data using Pymol software suite (https://pymol.org/2/#page-top).

Table S2: List of predicted antigenic peptide sequences in DIII domain. Immune epitope database (IEDB) analysis resource was used for the prediction on ZIKV DIII domain (residue range: 301KGVS….IGKA410) using Kolaskar and Tongaonkar algorithm. The antigenic propensity was computed on the overlapping heptapeptides. Residues from our energy calculations present in the predicted antigenic peptides are highlighted in red.

**Number**	**Start residue**	**End residue**	**Antigenic Peptide**	**length**
**1**	**304**	**311**	**SYSLCTAA**	**8**
**2**	**323**	**333**	**HGTVTVEVQYA**	**11**
**3**	**338**	**345**	**PCKVPAQM**	**8**
**4**	**352**	**358**	**LTPVGRL**	**7**
**5**	**384**	**391**	**DSYIVIGV**	**8**
